# Safety and tolerability of canakinumab, an IL-1β inhibitor, in type 2 diabetes mellitus patients: a pooled analysis of three randomised double-blind studies

**DOI:** 10.1186/1475-2840-13-94

**Published:** 2014-05-17

**Authors:** Campbell Howard, Adele Noe, Andrej Skerjanec, Björn Holzhauer, Margaret Wernsing, Monica Ligueros-Saylan, Tom Thuren

**Affiliations:** 1 Novartis Pharmaceuticals Corporation, USEH 100-214, One Health Plaza, East Hanover, NJ 07936-1080, USA; 2 Novartis Pharma AG, Basel, Switzerland

**Keywords:** Interleukin (IL)-1β inhibitor, Canakinumab, Safety, Type 2 diabetes mellitus

## Abstract

**Background:**

We aimed to assess the safety and tolerability of different doses of canakinumab versus placebo in patients with type 2 diabetes mellitus (T2DM).

**Methods:**

Data were pooled from three studies in 1026 T2DM patients with different routes of administration, treatment regimens and follow-up duration. Canakinumab groups were categorised as low (0.03 mg/kg i.v. once; N = 20), intermediate (0.1 and 0.3 mg/kg i.v. once, 5 and 15 mg s.c. monthly; N = 247), medium (1.5 mg/kg i.v. once, 50 mg s.c. monthly and 150 mg s.c. once; N = 268), and high doses (10 mg/kg i.v. once and 150 mg s.c. monthly; N = 137) and compared with placebo (N = 354). Incidences of adverse events (AEs), serious AEs (SAEs), discontinuations due to AEs, deaths, AEs of special interest related to interleukin-1β inhibition and T2DM disease, and laboratory abnormalities related to haematology and biochemistry parameters were reported. Safety was also analysed by age (<65, ≥65) and gender.

**Results:**

Average exposure across all groups was ≈ 6 months (maximum ~17 months). No dose response in AEs was observed but a trend towards more patients having at least one AE across canakinumab groups relative to placebo (P = 0.0152) was observed. SAEs were few and the incidence rate for most canakinumab groups was lower than that of placebo group except for the high-dose group (0.94% versus 0.58% per month in placebo). A total of five patients discontinued treatment due to AEs across treatment groups. No death was reported in any of the three studies. A small, non-significant increase in the incidence rate of infection AEs was observed on canakinumab groups relative to placebo. Canakinumab was associated with mostly mild decreases in WBC, neutrophils and platelet counts. Additionally, mild increases in SGPT, SGOT and bilirubin were reported. Overall, despite small differences, no clinically relevant findings were observed with respect to laboratory values and vital signs.

**Conclusions:**

This pooled analysis demonstrated that canakinumab was safe and well tolerated over a treatment period up to 1.4 years at the four pooled doses evaluated, in agreement with safety findings reported in the individual studies.

## Background

Inflammation plays a major role in all the stages of atherothrombosis, right from the initiation of endothelial injury to the formation of plaque/thrombus in the coronary arteries, which finally manifest into cardiovascular diseases such as acute myocardial infarction and stroke events [1,2]. Inflammation has also been recognised as an important contributor to β-cell dysfunction and apoptosis in type 2 diabetes mellitus (T2DM) patients [3-7]. Delay in progression of inflammation can help in the prevention of long-term cardiovascular risks.

Of the inflammatory molecules, interleukin-1 (IL-1) plays a major role in atherothrombosis [8-10]. Evidence supports the contribution of IL-1β isoform-mediated inflammation in atherogenesis and plaque progression, worsening of β-cell function in the islets, and disease progression [11-14].

One promising anti-inflammatory approach with potential relevance for cardiovascular disease is inhibition of the pro-inflammatory cytokine IL-1β. Canakinumab is a human monoclonal IL-1β antibody of the IgG1/k isotype that blocks the interaction of this cytokine with its receptors. This results in specific neutralisation of the bioactivity of IL-1β, but does not prevent the binding of the natural inhibitor IL-1Ra to the IL-1 receptor, nor the binding to IL-1α [15,16]. Canakinumab targets IL-1β–dependent inflammation, thereby potentially preserving/improving pancreatic β-cell function and inhibiting progression to atherothrombosis in the coronary arteries of T2DM patients who are at a high risk of developing cardiovascular disease. Canakinumab significantly reduces systemic high-sensitivity C-reactive protein (hsCRP), IL-6 and other inflammatory biomarker levels without tolerability issues [15,17-19]. Canakinumab is also being used to test the inhibitory effect on the inflammation in atherothrombosis in the ongoing Canakinumab ANti-inflammatory Thrombosis Outcome Study (CANTOS) trial. This randomised study will determine whether the long-term inhibition of IL-1β with canakinumab, as compared with placebo, reduces the rate of recurrent cardiovascular events among patients who are stable post-myocardial infarction but are at an increased cardiovascular risk due to increased levels of hsCRP (≥ 2 mg/L) despite usual care, including statin therapy [20].

In trials conducted to date, canakinumab has been associated with minimal injection-site reactions, and a few side effects due to IL-1β inhibition such as a small increase in the risk of infections, reflecting the inhibition of innate immunity achieved through canakinumab therapy. Infections were generally mild-to-moderate in severity, seldom serious and resolved spontaneously or with standard therapy [20].

On the basis of these considerations, it was of interest to assess the safety and tolerability of canakinumab, as well as the specific risk of adverse events (AEs) such as nasopharyngitis, urinary tract infections, thrombocytopenia and neutropenia as reported in previous studies [21-26]. We pooled data from two phase II and one phase IIb studies [24-27] that used different canakinumab doses. These studies were conducted in a relatively homogenous pool of patients comprising of T2DM and/or impaired glucose tolerance (IGT) patients. CANTOS is being conducted in post-myocardial infarction patients in whom T2DM is a common co-morbidity. Therefore, this integrated safety analysis in patients with T2DM and IGT aimed to provide additional support for the ongoing CANTOS trial with regard to patient safety.

## Methods

### Populations

The pooled safety analyses are based on previously published one phase IIb [24,25] and one phase II [26] studies, and reported one phase II study [27], in 1026 randomised patients. These studies were multicentre, randomised, double-blind, placebo-controlled studies comparing canakinumab with placebo in T2DM patients, on stable metformin monotherapy (except for one study [26] that comprised five strata: T2DM + metformin, T2DM + metformin/sulphonylurea, T2DM + metformin/sulphonylurea/thiazolidinedione, T2DM + insulin ± metformin and IGT patients). Both the phase II studies involved a single canakinumab injection, but the length of the subsequent follow-up differed [6 months follow-up in one, and 1 month full follow-up (i.e. laboratory, vital signs and all AEs) with an additional 3 months of follow-up only for SAEs in the other]. The phase IIb study had a monthly dosing schedule for a minimum of 4 months with all patients continuing monthly canakinumab treatment thereafter. This study had an interim efficacy analysis, when all the enrolled patients had completed 4 months of study drug, at which time the study was terminated due to a lack of sufficient short-term glucose lowering effect in T2DM patients.

### Assessments

Treatment-emergent AEs, serious AEs (SAEs), discontinuations due to AEs and deaths were assessed. Treatment-emergent AEs and SAEs were recorded and assessed by the investigator for their severity and possible relationship with the study medication. Biochemistry, haematological, lipid profile and urinalysis parameters were assessed. Patient demographics, vital signs and date of drug administration were also recorded.

### Data analysis

In order to pool data from different dosing regimens used in the three studies, the intravenous (i.v.) doses were converted to their subcutaneous (s.c.) equivalents by considering the 30% lower exposure with s.c. administration. Subsequently, doses were grouped according to cumulative and peak exposures and five different treatment groups were defined: placebo, low dose (0.03 mg/kg i.v. once), intermediate doses (0.1 and 0.3 mg/kg i.v. once, 5 and 15 mg s.c. monthly), medium doses (1.5 mg/kg i.v. once, 50 mg s.c. monthly and 150 mg s.c. once) and high doses (10 mg/kg i.v. once and 150 mg s.c. monthly).

Patient demographics, baseline characteristics, durations of exposure and follow-up were summarised. Owing to the long half-life of canakinumab (≈26 days), duration of exposure was defined as the time from first dose to last dose plus 3 months. The duration of follow-up was calculated as the time from first dose to the last follow-up visit plus 1 day.

AEs of special interest related to IL-1β inhibition and T2DM disease such as infections, malignancy risk, neutropenia, thrombocytopenia, hepatic disorders along with hypoglycaemia and major adverse cardiovascular events were analysed. AE coding for all three studies was updated to version 15.0 of the Medical Dictionary for Drug Regulatory Activities (MedDRA). AEs were coded to specific preferred terms (PTs), e.g., ‘Nasopharyngitis’, which may fall into one or more system organ classes (SOCs), e.g., ‘Infections and infestations’, of which one is considered the primary SOC and used for grouping PTs for reporting. To define the occurrence of AEs of special interest, current standardised MedDRA queries (SMQ) were used whenever they existed and were applied to the data, e.g., ‘Malignancies’ were defined by the narrow SMQ ‘Malignancies’. The criteria for identifying AEs of interest are provided in the online appendix (Additional file 1: Table S1). Laboratory abnormalities were classified by Common Toxicity Criteria (CTC) grades (grade 1–4).

To provide summary statistics for incidence of AEs that could be compared across studies of different lengths, the incidence rate (IR) per subject-month of follow-up was reported. The IR for AEs was defined as the total number of subjects with any AE divided by the total subject-months of follow-up until the first AE was reported or the end of follow-up. The IR for laboratory abnormalities was defined as subjects with an abnormality per number of post-baseline visits until the first abnormality occurred. The IRs for AEs and abnormal laboratory values are referred to as percentage (%) per month and % per visit, respectively, throughout the article.

Occurrence of binary outcomes such as AEs and laboratory abnormalities was meta-analysed across trials using Firth penalised likelihood logistic regression, with log length of follow-up as an offset variable and treatment and study as factors [28]. Continuous laboratory and vital signs data were meta-analysed using a repeated measures mixed effects model, with study, treatment, visit and visit by treatment interaction as factors, and baseline values as a covariate. Lipid profile parameters such as total cholesterol (TC), low-density lipoprotein (LDL) cholesterol, high-density lipoprotein (HDL) cholesterol and triglycerides (TG) were analysed on a log scale using the same approach, and the ratio of geometric means versus the placebo group was reported. P values and confidence intervals (CI) were interpreted in a purely descriptive manner due to lack of pre-specified hypotheses. Thus, any labelling of P values ≤0.05 as statistically significant has to be considered as purely hypothesis generating. All AE summaries were also analysed by age (<65, ≥65) and gender subgroups.

### Ethics and good clinical practice

Each of these studies (ClinicalTrials.gov Identifiers NCT00900146 [24,25], NCT01068860 [26] and NCT00900146/EUDRACT No. 2007-003729-26 [27]) was conducted as per the International Conference on Harmonization of Technical Requirements for Registration of Pharmaceuticals for Human Use (ICH) good clinical practice guidelines and in accordance with the ethical principles of the Declaration of Helsinki. All study participants provided written informed consent. All protocols were approved by the independent ethics committee/institutional review board at each study site or country.

## Results

### Exposure

The data on durations of exposure and follow-up across the three pooled clinical studies are provided in Table 1. The average exposure across all the groups was ≈ 6 months. In all treatment groups except the low-dose group, 25% of patients achieved an exposure of 7 to 9 months, with the maximum exposure being 16 to 17 months. Exposure was shorter in the low-dose and medium-dose groups due to the shorter planned duration of the studies involving these doses.

**Table 1:** Patient exposure and demographic and baseline characteristics by pooled dose

	**Canakinumab pooled dose**					
	**Low dose**	**Intermediate dose**	**Medium dose**	**High dose**	**Placebo**	**Total**
	**N = 20**	**N = 247**	**N = 268**	**N = 137**	**N = 354**	**N = 1026**
**Duration of exposure (months)**	3.00 ± 0.0	7.29 ± 3.057	4.93 ± 2.985	6.79 ± 3.119	5.82 ± 3.250	6.02 ± 3.235
≥3, n (%)	20 (100)	247 (100)	268 (100)	137 (100)	354 (100)	1026 (100)
≥4, n (%)	0	185 (74.9)	89 (33.2)	91 (66.4)	172 (48.6)	537 (52.3)
≥6, n (%)	0	179 (72.5)	87 (32.5)	90 (65.7)	166 (46.9)	522 (50.9)
≥8, n (%)	0	116 (47.0)	54 (20.1)	56 (40.9)	110 (31.1)	336 (32.7)
≥10, n (%)	0	37 (15.0)	19 (7.1)	15 (10.9)	35 (9.9)	106 (10.3)
Subject months of exposure	60.00	1800.61	1320.22	929.68	2067.64	6178.14
**Duration of follow-up (months)**	5.08 ± 1.426	6.34 ± 2.243	3.40 ± 3.056	6.43 ± 1.691	4.93 ± 2.898	5.07 ± 2.885
<1, n (%)	1 (5.0)	2 (0.8)	111 (41.4)	0	73 (20.6)	187 (18.2)
≥1, n (%)	19 (95.0)	245 (99.2)	157 (58.6)	137 (100)	281 (79.4)	839 (81.8)
≥2, n (%)	18 (90.0)	231 (93.5)	115 (42.9)	137 (100)	252 (71.2)	753 (73.4)
≥3, n (%)	18 (90.0)	228 (92.3)	114 (42.5)	136 (99.3)	251 (70.9)	747 (72.8)
≥4, n (%)	18 (90.0)	227 (91.9)	109 (40.7)	135 (98.5)	247 (69.8)	736 (71.7)
≥6, n (%)	0	123 (49.8)	59 (22.0)	62 (45.3)	115 (32.5)	359 (35.0)
≥8, n (%)	0	40 (16.2)	22 (8.2)	20 (14.6)	40 (11.3)	122 (11.9)
≥10, n (%)	0	12 (4.9)	7 (2.6)	5 (3.6)	15 (4.2)	39 (3.8)
Subject months of follow-up	101.58	1565.43	910.69	881.38	1744.57	5203.65
**Demographic variable**						
Age (years)	53.9 ± 9.07	54.4 ± 9.53	55.7 ± 10.05	54.9 ± 10.14	55.5 ± 9.65	55.2 ± 9.78
<65, n (%)	17 (85.0)	206 (83.4)	210 (78.4)	109 (79.6)	285 (80.5)	827 (80.6)
≥65, n (%)	3 (15.0)	41 (16.6)	58 (21.6)	28 (20.4)	69 (19.5)	199 (19.4)
Sex, n (%)						
Male	10 (50.0)	127 (51.4)	138 (51.5)	81 (59.1)	203 (57.3)	559 (54.5)
Female	10 (50.0)	120 (48.6)	130 (48.5)	56 (40.9)	151 (42.7)	467 (45.5)
Race, n (%)						
Caucasian	19 (95.0)	134 (54.3)	168 (62.7)	81 (59.1)	218 (61.6)	620 (60.4)
Black	1 (5.0)	12 (4.9)	8 (3.0)	5 (3.6)	14 (4.0)	40 (3.9)
Asian	0	81 (32.8)	77 (28.7)	41 (29.9)	97 (27.4)	296 (28.8)
Others	0	20 (8.1)	15 (5.7)	10 (7.3)	25 (7.0)	70 (6.9)
BMI (kg/m^2^), n (%)						
<30	9 (45.0)	131 (53.0)	115 (42.9)	72 (52.6)	183 (51.7)	510 (49.7)
≥30	11 (55.0)	116 (47.0)	153 (57.1)	65 (47.4)	171 (48.3)	516 (50.3)
HbA1c (%)	7.6 ± 0.79	7.5 ± 0.80	7.1 ± 0.74	7.6 ± 0.76	7.4 ± 0.85	7.4 ± 0.81
FPG (mmol/L)	8.8 ± 1.84	7.9 ± 1.87	7.7 ± 1.86	8.1 ± 1.56	8.0 ± 1.87	7.9 ± 1.83
Duration of type 2 diabetes (years)	5.0 ± 3.62	4.6 ± 4.64	7.0 ± 6.89	4.8 ± 4.70	5.6 ± 5.45	5.5 ± 5.59
Subjects with IGT, n	0	0	28	0	26	54
Diabetes complications, n (%)						
Retinopathy	NA	3 (1.6)	10 (4.6)	1 (1.1)	8 (3.3)	22 (3.0)
Neuropathy	NA	25 (13.3)	27 (12.4)	11 (12.0)	19 (7.8)	82 (11.1)
Nephropathy	NA	2 (1.1)	8 (3.7)	4 (4.3)	7 (2.9)	21 (2.8)
Use of statins, n (%)	2 (10.0)	64 (25.9)	104 (38.8)	32 (23.4)	87 (24.6)	289 (28.2)

Data are expressed as mean ± standard deviation (SD), unless otherwise stated.

*BMI* body mass index; *FPG* fasting plasma glucose; HbA1c, glycosylated haemoglobin A1c; IGT, impaired glucose tolerance.

Information on the use of statins and diabetes complications was not collected in one of the study (NA).

Low dose: 0.03 mg/kg i.v. once; intermediate dose: 0.1 and 0.3 mg/kg i.v. once, 5 and 15 mg s.c. monthly; medium dose: 1.5 mg/kg i.v. once, 50 mg s.c. monthly and 150 mg s.c. once; high dose: 10 mg/kg i.v. once and 150 mg s.c. monthly.

Duration of exposure (months) = (date of last dose–date of first dose + 91.2)/30.4.

Duration of follow-up (months) = (date of last follow-up visit–first dose date + 1)/30.4.

Subject months of exposure = sum of the duration of exposure (in months) over all subjects.

Subject months of follow-up = sum of the duration of follow-up (in months) over all subjects.

### Demography

The demographic and baseline characteristics by pooled doses are presented in Table 1. The demographic and baseline characteristics of patients were generally comparable across the treatment groups within each study and are summarised in online appendix (Additional file 2: Table S2). The overall mean age of patients was approximately 55 years with 19.4% of patients aged ≥65 years. Proportion of men and women was almost equal in most treatment groups, with a slightly higher percentage of men in the high-dose group and the placebo group. Patients were predominantly of Caucasian race across groups. The overall mean HbA1c value of the study population was 7.4% with slightly lower values in the medium-dose canakinumab group (7.1%), which included IGT patients unlike the other canakinumab groups. Distribution of patients with the body mass index (BMI) <30 kg/m^2^ and ≥30 kg/m^2^ was almost similar in most treatment groups. Mean duration of T2DM was approximately 5 years, except for the medium-dose group with 7 years. This is because one study of the medium dose versus placebo allowed the inclusion of patients receiving multiple oral drugs with or without insulin, which typically reflects advanced disease stages. The proportion of patients using statins was 20% to 40% in all groups, except the low-dose group at 10%. Diabetic neuropathy was a more common complication among patients across groups than retinopathy or nephropathy.

### Safety and tolerability

Overall, incidences of any AEs, any SAEs and discontinuations due to any AEs are summarised in Table 2. A trend towards more patients having at least one AE across canakinumab doses relative to placebo (P = 0.0152) was seen. SAEs were few and the IR was lower in most of the canakinumab groups compared with the placebo group, except in the high-dose group (0.94% versus 0.58% per month for placebo). There were no SAEs reported in patients receiving low-dose canakinumab. Treatment discontinuation due to AEs was reported in five patients across groups. There were no deaths reported in any of the studies.

**Table 2:** Summary of adverse events reported in the pooled data

	**Canakinumab pooled dose**				**Placebo**
	**Low dose**	**Intermediate dose**	**Medium dose**	**High dose**	
	**N = 20**	**N = 247**	**N = 268**	**N = 137**	**N = 354**
**Any AEs**					
n (%)	10 (50.0)	107 (43.3)	103 (38.4)	67 (48.9)	134 (37.9)
IR (%)	16.31	10.28	16.45	12.14	11.45
**Discontinuations due to any AEs**					
n (%)	1 (5.0)	1 (0.4)	0	2 (1.5)	1 (0.3)
IR (%)	0.99	0.06		0.23	0.06
**Any serious AEs**					
n (%)	0	4 (1.6)	5 (1.9)	8 (5.8)	10 (2.8)
IR (%)	0	0.26	0.55	0.94	0.58
**Deaths**					
n (%)	0	0	0	0	0
**AEs by primary SOCs (occurring in ≥5% of patients in any group)**					
Infections and infestations					
n (%)	4 (20.0)	54 (21.9)	40 (14.9)	30 (21.9)	49 (13.8)
IR (%)	4.41	4.05	5.13	4.02	3.17
Musculoskeletal and connective tissue disorders					
n (%)	1 (5.0)	22 (8.9)	14 (5.2)	11 (8.0)	27 (7.6)
IR (%)	1.03	1.48	1.62	1.33	1.65
Gastrointestinal disorders					
n (%)	1 (5.0)	12 (4.9)	17 (6.3)	12 (8.8)	21 (5.9)
IR (%)	1.04	0.80	1.98	1.44	1.26
Cardiac disorders					
n (%)	1 (5.0)	8 (3.2)	4 (1.5)	3 (2.2)	12 (3.4)
IR (%)	1.04	0.53	0.44	0.35	0.71
Eye disorders					
n (%)	3 (15.0)	5 (2.0)	6 (2.2)	2 (1.5)	7 (2.0)
IR (%)	3.40	0.33	0.67	0.23	0.41
Renal and urinary disorders					
n (%)	1 (5.0)	5 (2.0)	5 (1.9)	5 (3.6)	3 (0.8)
IR (%)	1.04	0.32	0.55	0.58	0.17
Investigations					
n (%)	0	12 (4.9)	13 (4.9)	16 (11.7)	15 (4.2)
IR (%)		0.79	1.50	1.96	0.89
Skin and subcutaneous tissue disorders					
n (%)	0	10 (4.0)	11 (4.1)	7 (5.1)	16 (4.5)
IR (%)		0.65	1.25	0.83	0.95

n (%) are expressed as number (%) of patients; incidence rate (IR) is expressed as % per month.

Low dose: 0.03 mg/kg i.v. once; intermediate dose: 0.1 and 0.3 mg/kg i.v. once, 5 and 15 mg s.c. monthly; medium dose: 1.5 mg/kg i.v. once, 50 mg s.c. monthly and 150 mg s.c. once; high dose: 10 mg/kg i.v. once and 150 mg s.c. monthly.

*SOC* system organ class.

#### Adverse events

When the reported AEs were analysed by primary SOC (Table 2), there were no major imbalances in IRs between the canakinumab and placebo groups. A slightly higher IR of AEs was reported in canakinumab-treated patients compared with placebo under the SOCs of infections and infestations, gastrointestinal, renal and urinary disorders and investigations. Conversely, a slightly lower incidence of AEs was reported in canakinumab-treated patients compared with placebo under the SOCs of cardiac disorders (except low-dose group) and skin and subcutaneous tissue disorders (except medium-dose group). Table 3 provides a summary of the most commonly reported AEs by preferred terms (occurring in at least five patients and ≥2% in any of the group). The most common AE preferred terms reported across treatment groups were nasopharyngitis, bronchitis, urinary tract infection, upper respiratory tract infection and headache. The IR of urinary tract infection AEs was lower across canakinumab doses compared with the placebo group with the exception of the low-dose canakinumab group. The IRs of upper respiratory tract infection appeared slightly higher for all canakinumab doses—except for the low dose—relative to placebo. There was no imbalance between canakinumab doses and the placebo group for nasopharyngitis, arthralgia, pain in extremity and headaches.

**Table 3:** Incidence of most common adverse events (occurring in at least five patients and in ≥2% in any group) by preferred term

**Preferred term**	**Canakinumab pooled dose**				**Placebo**
	**Low dose**	**Intermediate dose**	**Medium dose**	**High dose**	
	**N = 20**	**N = 247**	**N = 268**	**N = 137**	**N = 354**
Nasopharyngitis					
n (%)	0	15 (6.1)	10 (3.7)	11 (8.0)	21 (5.9)
IR (%)		1.00	1.15	1.31	1.26
Bronchitis					
n (%)	0	9 (3.6)	3 (1.1)	1 (0.7)	2 (0.6)
IR (%)		0.59	0.33	0.11	0.11
Urinary tract infection					
n (%)	3 (15.0)	7 (2.8)	5 (1.9)	5 (3.6)	11 (3.1)
IR (%)	3.15	0.46	0.56	0.58	0.64
Upper respiratory tract infection					
n (%)	0	6 (2.4)	3 (1.1)	4 (2.9)	4 (1.1)
IR (%)		0.39	0.33	0.47	0.23
Arthralgia					
n (%)	0	5 (2.0)	3 (1.1)	3 (2.2)	7 (2.0)
IR (%)		0.32	0.33	0.35	0.41
Pain in extremity					
n (%)	0	5 (2.0)	4 (1.5)	3 (2.2)	5 (1.4)
IR (%)		0.32	0.44	0.35	0.29
Headache					
n (%)		4 (1.6)	2 (0.7)	5 (3.6)	8 (2.3)
IR (%)		0.26	0.22	0.58	0.47
Cough					
n (%)		2 (0.8)	3 (1.1)	2 (1.5)	7 (2.0)
IR (%)		0.13	0.33	0.23	0.41
Oropharyngeal pain					
n (%)		5 (2.0)	0	2 (1.5)	0
IR (%)		0.32		0.23	

n (%) are expressed as number (%) of patients; incidence rate (IR) is expressed as % per month.

Low dose: 0.03 mg/kg i.v. once; intermediate dose: 0.1 and 0.3 mg/kg i.v. once, 5 and 15 mg s.c. monthly; medium dose: 1.5 mg/kg i.v. once, 50 mg s.c. monthly and 150 mg s.c. once; high dose: 10 mg/kg i.v. once and 150 mg s.c. monthly.

#### Serious adverse events

There were no appreciable trends in SAEs observed, and the majority of SAEs were scattered across different SOCs (Table 4). According to the primary SOC, the highest incidence of SAEs across different groups was ‘infections and infestations’ with a higher IR for the high-dose canakinumab group (three patients, IR = 0.34% per month) compared with placebo (two patients, IR = 0.12% per month). Canakinumab was associated with a lower IR of cardiac SAEs than placebo (high dose: one patient, IR = 0.11% per month; placebo: five patients, IR = 0.29% per month).

**Table 4:** Incidence of serious adverse events by primary SOC

	**Canakinumab pooled dose**				**Placebo**
	**Low dose**	**Intermediate dose**	**Medium dose**	**High dose**	
	**N = 20**	**N = 247**	**N = 268**	**N = 137**	**N = 354**
Infections and infestations					
n (%)	0	1 (0.4)	1 (0.4)	3 (2.2)	2 (0.6)
IR (%)		0.06	0.11	0.34	0.12
Renal and urinary disorders					
n (%)	0	1 (0.4)	2 (0.7)	1 (0.7)	1 (0.3)
IR (%)		0.06	0.22	0.11	0.06
Gastrointestinal disorders					
n (%)	0	0	1 (0.4)	1 (0.7)	1 (0.3)
IR (%)			0.11	0.11	0.06
Cardiac disorders					
n (%)	0	0	0	1 (0.7)	5 (1.4)
IR (%)				0.11	0.29
Hepatobiliary disorders					
n (%)	0	0	1 (0.4)	0	1 (0.3)
IR (%)			0.11		0.06
General disorders and administration site conditions					
n (%)	0	0	0	0	1 (0.3)
IR (%)					0.06
Musculoskeletal and connective tissue disorders					
n (%)	0	1 (0.4)	0	0	1 (0.3)
IR (%)		0.06			0.06
Metabolism and nutrition disorders					
n (%)	0	0	0	2 (1.5)	0
IR (%)				0.23	
Investigations					
n (%)	0	0	0	1 (0.7)	0
IR (%)				0.11	
Neoplasms—benign, malignant and unspecified (including cysts and polyps)					
n (%)	0	0	0	1 (0.7)	0
IR (%)				0.11	
Injury, poisoning and procedural complications					
n (%)	0	0	1 (0.4)	0	0
IR (%)			0.11		
Nervous system disorders					
n (%)	0	1 (0.4)	0	0	0
IR (%)		0.06			
Reproductive system and breast disorders					
n (%)	0	1 (0.4)	0	0	0
IR (%)		0.06			

n (%) are expressed as number (%) of patients; incidence rate (IR) is expressed as % per month.

Low dose: 0.03 mg/kg i.v. once; intermediate dose: 0.1 and 0.3 mg/kg i.v. once, 5 and 15 mg s.c. monthly; medium dose: 1.5 mg/kg i.v. once, 50 mg s.c. monthly and 150 mg s.c. once; high dose: 10 mg/kg i.v. once and 150 mg s.c. monthly.

#### Adverse events of special interest

AEs of special interest including those potentially related to IL-1β inhibition and T2DM disease are presented in Table 5. Although the rate of infections was numerically higher in all canakinumab groups (4.41%, 4.05%, 5.13% and 4.02% per month in low, intermediate, medium and high doses, respectively) than placebo (3.17% per month), the difference was not statistically significant—neither for each individual dose nor when tested for a monotonic trend across doses versus placebo (P = 0.1255). Infections were primarily recorded due to unspecified pathogens affecting the upper and lower respiratory tract, other infections were recorded as viral or bacterial infections. Hypersensitivity/allergy IRs was lower in all canakinumab doses compared with placebo with the exception of the medium dose (1.02% versus 0.84% per month). The IR of thrombocytopenia AEs were higher across all canakinumab groups (1.04%, 0.46%, 0.56% and 0.46% per month in low-, intermediate-, medium- and high-dose groups, respectively) compared with placebo (0.23% per month). There was no imbalance in the IR of hypertension AEs between the canakinumab and placebo groups. Major adverse cardiovascular events occurred in patients of all groups, except the low-dose group; however, incidences in the intermediate-, medium- and high-dose groups were lower versus the placebo group (0.19%, 0.44% and 0.23% versus 0.53% per month, respectively). This observed difference in favour of canakinumab was not statistically significant—neither for each individual dose nor when tested for a monotonic trend across doses versus placebo (P = 0.9371).

**Table 5:** Adverse events of special interest including class effect (IL-1β inhibition) and disease effect (T2DM)

	**Canakinumab pooled dose**				**Placebo**
	**Low dose**	**Intermediate dose**	**Medium dose**	**High dose**	
	**N = 20**	**N = 247**	**N = 268**	**N = 137**	**N = 354**
Infections					
n (%)	4 (20.0)	54 (21.9)	40 (14.9)	30 (21.9)	49 (13.8)
IR (%)	4.41	4.05	5.13	4.02	3.17
Hypersensitivity/allergy					
n (%)	0	9 (3.6)	9 (3.4)	6 (4.4)	14 (4.0)
IR (%)	0	0.59	1.02	0.70	0.84
Thrombocytopenia					
n (%)	1 (5.0)	7 (2.8)	5 (1.9)	4 (2.9)	4 (1.1)
IR (%)	1.04	0.46	0.56	0.46	0.23
Hypertension					
n (%)	0	5 (2.0)	3 (1.1)	3 (2.2)	6 (1.7)
IR (%)	0	0.32	0.33	0.35	0.35
Major adverse cardiovascular events (MACE)					
n (%)	0	3 (1.2)	4 (1.5)	2 (1.5)	9 (2.5)
IR (%)	0	0.19	0.44	0.23	0.53
Hepatic disorders					
n (%)	0	1 (0.4)	2 (0.7)	6 (4.4)	2 (0.6)
IR (%)	0	0.06	0.22	0.70	0.12
Vertigo					
n (%)	0	1 (0.4)	5 (1.9)	4 (2.9)	6 (1.7)
IR (%)	0	0.06	0.56	0.46	0.3
Injection-site reactions					
n (%)	0	0	2 (0.7)	0	2 (0.6)
IR (%)	0	0	0.22	0	0.12
Hypoglycaemia					
n (%)	0	0	2 (0.7)	0	1 (0.3)
IR (%)	0	0	0.22	0	0.06
Changes in renal function					
n (%)	0	1 (0.4)	1 (0.4)	0	0
IR (%)	0	0.06	0.11	0	0
Lymphoid organ toxicity					
n (%)	0	0	1 (0.4)	0	0
IR (%)	0	0	0.11	0	0
Malignancies					
n (%)	0	0	0	1 (0.7)	0
IR (%)	0	0	0	0.11	0
Neutropenia					
n (%)	0	1 (0.4)	0	0	0
IR (%)	0	0.06	0	0	0

n (%) are expressed as number (%) of patients; incidence rate (IR) is expressed as % per month.

Low dose: 0.03 mg/kg i.v. once; intermediate dose: 0.1 and 0.3 mg/kg i.v. once, 5 and 15 mg s.c. monthly; medium dose: 1.5 mg/kg i.v. once, 50 mg s.c. monthly and 150 mg s.c. once; high dose: 10 mg/kg i.v. once and 150 mg s.c. monthly.

The IR of hepatic disorders AEs was higher in the high-dose canakinumab (0.70% per month) than in the other canakinumab groups (0%, 0.06% and 0.22% per month for low, intermediate and medium groups, respectively) or placebo (0.12% per month), with an odds ratio versus placebo of 6.217 (95% CI: 1.504–25.697; P = 0.0116). Six patients in the high-dose group reported events, mainly hepatic steatosis and abnormality in hepatic enzymes levels such as aspartate aminotransferase (AST), aspartate alanine transferase (ALT) elevations, but none of abnormalities based on the central laboratory exceeded >3 times upper limit of normal (ULN) range.

Injection-site reactions and hypoglycaemic events were only reported in patients who received medium dose of canakinumab or placebo with slightly higher rates in the medium dose than placebo (0.22% versus 0.12% per month and 0.22% versus 0.06% per month, respectively). AEs of lymphoid organ toxicity, malignancies and neutropenia were only observed in one patient for each event and changes in renal function were observed in two patients receiving canakinumab.

#### Haematology and biochemistry abnormalities

**Figure 1:**
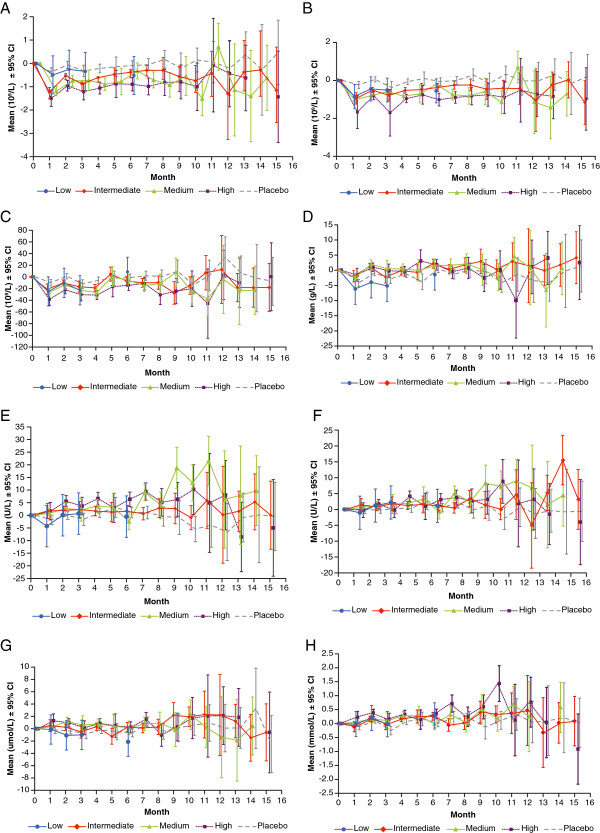
**Model-adjusted change from baseline in haematological and biochemistry parameters, by time point. A)**. White blood cell counts (total) (10^9^/L). **B)**. Absolute neutrophil counts (10^9^/L). **C)**. Platelet count (direct) (10^9^/L). **D)**. Haemoglobin (g/L). **E)**. Serum glutamic pyruvic transaminase/ALT (U/L). **F)**. Serum glutamic oxaloacetic transaminase/AST (U/L). **G)**. Bilirubin (total) (μmol/L). **H)**. Cholesterol (total) (mmol/L). *Low dose: 0.03 mg/kg i.v. once; intermediate dose: 0.1 and 0.3 mg/kg i.v. once, 5 and 15 mg s.c. monthly; medium dose: 1.5 mg/kg i.v. once, 50 mg s.c. monthly and 150 mg s.c. once; high dose: 10 mg/kg i.v. once and 150 mg s.c. monthly.*

Specified abnormalities in haematology and biochemistry parameters are summarised in Table 6 and treatment group averages over time are presented in Figure 1. Decrease in total WBC counts was observed on canakinumab (Figure 1A) with no clear dose-related pattern. Patients receiving intermediate, medium and high doses of canakinumab had a higher IR of abnormally low total WBC (2.14%, 3.22% and 1.86% per visit, respectively) than the placebo group (1.25% per visit). Most of these abnormalities were grade 1. Only two patients (one each in intermediate and placebo groups) had a grade 3 abnormality. There was no clear association between patients with abnormal WBC counts and those who had infections. There was also a decrease in absolute neutrophil counts (Figure 1B) with a higher IR of abnormal neutrophil counts in patients receiving intermediate (2.68% per visit), medium (4.95% per visit) and high doses (4.61% per visit) than in those receiving low-dose canakinumab (0.89% per visit) or placebo (1.22% per visit). The difference was statistically significant for each individual dose or when tested for a monotonic trend across doses versus placebo (P = 0.0001). No CTC grade 3 or 4 abnormality was observed. There was no clear association between patients with abnormal neutrophil counts and those who had infections. There was also a decrease in mean platelet counts (Figure 1C) with no clear dose-related pattern. Patients receiving intermediate and medium doses showed similar IRs of platelet count abnormality compared with placebo (1.23%, 1.57% versus 1.00% per visit, respectively), whereas lower IRs were reported for the low and high canakinumab doses relative to placebo (0.92%, 0.73% versus 1.00% per visit, respectively). Of note, this decrease in platelet counts in canakinumab-treated patients versus placebo was no longer apparent after 5 months. Most of the abnormalities in platelet count reported were grade 1 or 2 with a comparable IR as in the placebo group. No patients with abnormal platelet counts had a bleeding disorder. No difference in the IR for abnormal absolute lymphocyte counts was seen between the canakinumab and placebo groups. Data show a trend towards higher haemoglobin levels in canakinumab groups compared with placebo, which persisted for at least 5 months of treatment (Figure 1D).

**Table 6:** Number of subjects (incidence rates) with specified abnormalities in haematology and biochemistry parameters during any time of the post-treatment follow-up period

	**Canakinumab pooled dose**				**Placebo**
	**Low dose**	**Intermediate dose**	**Medium dose**	**High dose**	
**I. Haematology parameters**					
WBC (total) (10^9^/L), N	20	245	262	136	342
Any grade 1–4 abnormality, n	0	19	18	10	14
IR (%)	0.0	2.14	3.22	1.86	1.25
Dose trend test, P value			0.0884		
G1 (<LLN–3.0), n (%)	0	18 (7.3)	15 (5.7)	10 (7.4)	13 (3.8)
IR (%)	0.0	2.03	2.68	1.86	1.16
G2 (<3.0–2.0), n (%)	0	1 (0.4)	4 (1.5)	1 (0.7)	1 (0.3)
IR (%)	0.0	0.11	0.68	0.18	0.09
Absolute neutrophil counts (Seg. + bands) (10^9^/L), N	20	222	258	93	288
Any grade 1–4 abnormality, n	1	21	27	13	10
IR (%)	0.89	2.68	4.95	4.61	1.22
Dose trend test, P value			0.0001		
G1 (<LLN–1.5), n (%)	0	17 (7.7)	23 (8.9)	13 (14.0)	7 (2.4)
IR (%)	0.0	2.13	4.17	4.59	0.85
G2 (<1.5–1.0), n (%)	1 (5.0)	6 (2.7)	7 (2.7)	2 (2.2)	2 (0.7)
IR (%)	0.89	0.74	1.26	0.66	0.24
Platelet counts (direct) (10^9^/L), N	20	245	260	136	342
Any grade 1–4 abnormality, n	1	11	9	4	11
IR (%)	0.92	1.23	1.57	0.73	1.00
Dose trend test, P value			0.9488		
G1 (<LLN–75), n (%)	1 (5.0)	10 (4.1)	9 (3.5)	4 (2.9)	11 (3.2)
IR (%)	0.92	1.11	1.57	0.73	1.00
G2 (<75–50), n (%)	0	0	0	1 (0.7)	0
IR (%)	0.0	0.0	0.0	0.18	0.0
Absolute lymphocyte counts (10^9^/L), N	20	222	258	93	288
Any grade 1–4 abnormality, n	0	2	3	1	7
IR (%)	0.0	0.25	0.53	0.33	0.85
Dose trend test, P value			0.2241		
G1 (<LLN–0.8), n (%)	0	0	1 (0.4)	0	3 (1.0)
IR (%)	0.0	0.0	0.18	0.0	0.36
G2 (<0.8–0.5), n (%)	0	2 (0.9)	2 (0.8)	1 (1.1)	4 (1.4)
IR (%)	0.0	0.25	0.36	0.33	0.48
Haemoglobin (g/L), N	20	245	262	136	342
Any grade 1–4 abnormality, n	8	49	36	31	76
IR (%)	9.20	6.27	6.83	6.68	7.97
Dose trend test, P value			0.1239		
G1 (<LLN–100), n (%)	8 (40.0)	49 (20.0)	33 (12.6)	30 (22.1)	73 (21.3)
IR (%)	9.09	6.23	6.24	6.41	7.63
G2 (<100–80), n (%)	1 (5.0)	5 (2.0)	3 (1.1)	2 (1.5)	5 (1.5)
IR (%)	0.90	0.54	0.51	0.36	0.44
**II. Biochemistry parameters**					
SGPT (ALT) (U/L), N	20	245	262	136	343
Any grade 1–4 abnormality, n	0	2	4	5	2
IR (%)	0.0	0.21	0.69	0.88	0.17
Dose trend test, P value			0.0158		
G1 (>3–5×ULN), n (%)	0	2 (0.8)	4 (1.5)	5 (3.7)	2 (0.6)
IR (%)	0.0	0.21	0.69	0.88	0.17
SGOT (AST) (U/L), N	20	245	256	136	342
Any grade 1–4 abnormality, n	0	1	0	2	0
IR (%)	0.0	0.11	0.0	0.35	0.0
Dose trend test, P value			0.0777		
G1 (>3–5×ULN), n (%)	0	1 (0.4)	0	2 (1.5)	0
IR (%)	0.0	0.11	0.0	0.35	0.0
Bilirubin (total) (μmol/L), N	20	245	265	136	344
Any grade 1–4 abnormality, n	0	0	3	4	6
IR (%)	0.0	0.0	0.52	0.70	0.52
Dose trend test, P value			0.6430		
G1 (>1.5–2×ULN), n (%)	0	0	2 (0.8)	3 (2.2)	5 (1.5)
IR (%)	0.0	0.0	0.34	0.52	0.44
G2 (>2–3×ULN), n (%)	0	0	2 (0.8)	2 (1.5)	0
IR (%)	0.0	0.0	0.34	0.35	0.0
Alkaline phosphatase, serum (U/L), N	20	245	263	136	344
Any grade 1–4 abnormality, n	0	0	0	0	1
IR (%)	0.0	0.0	0.0	0.0	0.09
Dose trend test, P value			0.2357		
G1 (>2–3×ULN), n (%)	0	0	0	0	1 (0.3)
IR (%)	0.0	0.0	0.0	0.0	0.09
G2 (>3–5×ULN), n (%)	0	0	0	0	1 (0.3)
IR (%)	0.0	0.0	0.0	0.0	0.09
Creatinine (umol/L), N	20	245	265	136	344
Any grade 1–4 abnormality, n	1	10	11	6	10
IR (%)	0.91	1.06	1.89	1.06	0.88
Dose trend test, P value			0.3490		
G1 (>ULN–1.5×ULN), n (%)	1 (5.0)	10 (4.1)	11 (4.2)	6 (4.4)	10 (2.9)
IR (%)	0.91	1.06	1.88	1.06	0.88
G2 (>1.5–3×ULN), n (%)	0	1 (0.4)	1 (0.4)	0	0
IR (%)	0.0	0.11	0.17	0.0	0.0

Abnormalities are presented as number (n) and/or % of patients. Incidence rate (IR) is expressed as % per visit. Subjects are counted only once if specified abnormality occurred multiple times during the treatment or post-treatment follow-up period. Subjects can be counted more than once in multiple categories and grades. The Jonckheere-Terpstra test was used to test for a monotonic trend across doses in the proportion of post-baseline visits with any laboratory abnormality among the total number of post-baseline visits.

CTC, common terminology criteria; G, grade; WBC, white blood cell; SGOT, serum glutamic oxaloacetic transaminase; SGPT, serum glutamic pyruvic transaminase.

Low dose: 0.03 mg/kg i.v. once; intermediate dose: 0.1 and 0.3 mg/kg i.v. once, 5 and 15 mg s.c. monthly; medium dose: 1.5 mg/kg i.v. once, 50 mg s.c. monthly and 150 mg s.c. once; high dose: 10 mg/kg i.v. once and 150 mg s.c. monthly.

Total WBC count: Normal range = 4.5–11.0 × 10^9^/L; Absolute neutrophil counts: Normal range = 1.5–8.0 × 10^9^/L; Haemoglobin: Normal range = 13.5–17.5% (males) and 12.0–16.0% (females); Platelet count (direct): Normal range = 150–450 × 10^9^/L; Absolute lymphocyte count: Normal range = 1.0–4.0 × 10^9^/L; SGOT (AST): Normal range = 3–44 U/L; SGPT (ALT): Normal range = 0–40 U/L; Bilirubin (total): Normal range = 5–17.0 μmol/L; Alkaline phosphatase, serum: Normal range = 30–125 U/L; Creatinine: Normal range = 45–90 μmol/L (females) and 60–110 μmol/L (males).

Increases in mean SGPT (ALT) levels were noted, which did not worsen over time (Figure 1E). These increases were less discernible in SGOT (AST) levels (Figure 1F). The IRs of elevations in hepatic enzymes CTC grade 1 were 0%, 0.21%, 0.69%, 0.88% and 0.17% per visit for SGPT and 0%, 0.11%, 0%, 0.35% and 0% per visit for SGOT in the low-, intermediate-, medium-, high-doses and placebo groups, respectively. There was also an increase in mean levels of total bilirubin, which were, however, no longer discernible after 7 months of treatment (Figure 1G). There was no difference in incidence of elevated bilirubin levels reported in medium- and high-dose groups versus placebo. The reported abnormalities were of grade 1 or 2 except one patient in the placebo group who had grade 3 abnormality. The monotonic trend across doses was statistically significant versus placebo (P = 0.0158) for SGPT abnormalities, whereas this was not the case for SGOT and bilirubin levels. There were no potential Hy’s Law cases (ALT or AST ≥3xULN and total bilirubin >2xULN) reported in any of the three studies.

An increase in average TC (medium dose: 3%, 2%, 8% and 4%; high dose: 7%, 5%, 8% and 5% for Months 1 to 4, Figure 1H) and TG (high dose: 11%, 15%, 14% and 10% for Months 1 to 4) levels was seen in patients treated with medium and/or high doses of canakinumab for at least the initial 4 months compared with placebo. Average HDL-C was higher in the high dose (5% for Month 1) and medium dose (7% and 5% for Months 1 and 2) than placebo during the first 2 months of treatment. No clear time pattern was identified for LDL-C.

#### Changes in vital signs

Modest increases from baseline in systolic and diastolic blood pressures were generally observed in the canakinumab groups throughout the duration of the observation period. However, the majority of these changes in either systolic or diastolic blood pressure were not significantly different from those seen in placebo and had no particular dose-related or temporal trend. There was no clear dose response or trend seen in changes in the pulse rate in patients treated with canakinumab. No considerable change in body weight was observed across groups.

#### Sub group analyses by age and gender

Total AEs, SAEs, haematology and biochemistry parameters were analysed by age (<65 and ≥65 years) and gender versus placebo and are summarised in online appendix (Additional file 3: Table S3). Findings in age subgroups were generally similar to those in the overall population with no notable differences in the safety profile of canakinumab. Women treated with canakinumab reported numerically higher incidences of any AEs than women in the placebo group. In contrast, incidences of any AEs in men treated with canakinumab were lower than those treated with placebo, except in patients receiving intermediate dose who showed a slight increase in AEs compared with the placebo group. However, the incidences of any SAEs in men and women were generally comparable with placebo and no notable differences to the overall population were observed with respect to laboratory values and vital signs.

## Discussion

Inflammation plays a major role in endothelial dysfunction and the development of atherothrombosis. There are several novel targets available to inhibit these inflammatory changes at the vascular level such as tumor necrosis factor-α (TNF-α) blocker (etanercept, rilonacept, infliximab, adalimumab), IL-6 receptor antagonist (tocilizumab), IL-1a receptor antagonist (anakinra) and IL-1β (canakinumab) [29]; and steroidogenic acute regulatory protein, so far studied only in rats [30]. Targeting of the IL-1 pathways in disease treatment raises possible safety concerns due to inhibition of innate immunity [31]. This large pooled analysis included more than 1000 patients with type 2 diabetes mellitus (mean duration: ~5 years) treated for up to 1.4 years either with canakinumab, an IL-1β blocker or placebo. It was of particular interest to assess the combined safety data from individual studies to help identify any significant findings, including dose response, from the pooled analysis.

Overall, there were no clinically meaningful differences in AEs and SAEs between canakinumab-treated patients and placebo, with no clear dose-dependent trends in AEs. The observed safety findings and overall tolerability in patients with T2DM are consistent with those in previously reported studies in patients with cryopyrin-associated periodic syndrome, gouty arthritis and systemic juvenile idiopathic arthritis [21-23].

The mode of action of canakinumab is to inhibit innate immunity while binding to IL-1β [31], therefore, an increased rate of infections was expected in each canakinumab dose group versus placebo. The most commonly reported AEs in this pooled analyses were nasopharyngitis, urinary tract infections and upper respiratory tract infections. The observed increase in rate of infections with canakinumab was, however, not statistically significant. This numerical imbalance is, nevertheless, most likely a true signal due to its biological plausibility. Importantly, the clinical experience with canakinumab differs from that of TNF-α blockers which are associated with an increased risk of tuberculosis reactivation [32]. Studies reveal that tuberculosis risk varies with the use of different anti-TNF-α agents (etanercept, 35 cases per 100,000 patients; infliximab, 144 per 100,000 patients; and adalimumab, 240 per 100,000 patients) [33]. Wallis et al. [34] showed that the probability of tuberculosis developing with infliximab treatment is threefold higher than with etanercept. Similar to anakinra [35], canakinumab has not been associated with the risk of tuberculosis reactivation, indicating that blocking the IL-1 pathway is safer in comparison with blocking TNF-α.

Of the seven patients who experienced serious infections, five were observed on canakinumab and two on placebo. Four of the five canakinumab-treated subjects had a complete recovery and in one case outcome was not reported after the patient was taken off the study drug. No relationship between the events and study drug was reported by the investigators for the patients who reported an outcome. Moreover, there were no significant changes in the WBC counts in these seven patients, with the only value falling below the normal range at month 4 (3.12×10^9^/L; baseline 7.13×10^9^/L) in a patient taking placebo which returned to normal range at month 6 (5.38×10^9^/L). Although none of the patients had WBC values above the upper limit of normal range throughout the study, two patients reported leucocytosis associated with their acute infection. There were no significant changes in hsCRP levels in these patients.

AEs of injection-site reactions, malignancies, neutropenia and changes in renal function were infrequent with no apparent dose-dependent trend. Although IL-1β inhibition increases expression of the pancreatic β-cells in T2DM patients [13], the present study reported only two hypoglycaemic events with medium-dose canakinumab (versus one on placebo), which were mild-to-moderate in severity and none was suspected by the investigators to be related to the study drug. None of the patients had reported any asymptomatic low blood glucose (<40 mg/dL) event or discontinued their study treatment permanently because of any hypoglycaemic event, thereby suggesting that canakinumab did not result in increased risk of hypoglycaemic events in T2DM patients. Although a lower rate of major adverse cardiovascular events was observed in the canakinumab groups compared with the placebo group, the low number of events prevents drawing conclusions on the significance of this observation.

The decreases in platelet counts and absolute neutrophil counts and the resulting decreased total WBC counts are well-known effects of canakinumab treatment [21-23] due to the role of IL-1β in bone marrow maturation and release of these blood cells [31]. Most laboratory abnormalities observed in this pooled analysis were of CTC grades 1 or 2, and there was no association of any such events either with infections or bleeding/bruising.

The incidence rate of elevations of hepatic enzymes was low and was driven by a small number of patients with a known high prevalence of hepatic steatosis. There were no cases consistent with Hy’s law. Vital signs of the patients treated with canakinumab showed no clinically meaningful difference versus placebo and had no particular dose-related trend. Treatment with canakinumab did not seem to have a clinically meaningful effect on lipid profiles in patients with T2DM or IGT. The increase in TC levels appeared to be due to an increase in HDL-C, because no clear pattern was observed for LDL-C. The clinical significance of the increase in TG levels could not be determined in the absence of increase in LDL-C levels.

Treatment-emergent AEs leading to permanent discontinuation of study treatment were rare and evenly distributed in all treatment groups, demonstrating the good tolerability and safety of canakinumab. No deaths were reported in any of the three studies. The subgroup analyses by age (<65 and ≥65 years) and gender showed no remarkable observations related to the safety findings of canakinumab when compared with overall population. Safety findings from this pooled data set were consistent with the previously reported studies in patients with cryopyrin-associated periodic syndrome, gouty arthritis and systemic juvenile idiopathic arthritis [21-23] with no new safety findings. In addition, a recent study in newly diagnosed type 1 diabetes patients (mostly less than 18 years of age) treated with IL-1Ra (anakinra) and IL-1β (canakinumab) inhibitors revealed no major safety concerns although they failed to prevent deterioration of endogenous pancreatic insulin (C-peptide) secretion in these patients [36]. However, in a preclinical study [37], the combination of IL-1β blocker with other anti-CD3 monoclonal antibody resulted in significantly greater clinical remission of diabetes suggesting that this combination might be more suitable in new onset type 1 diabetes or in prevention trials in individuals with pre-type 1 diabetes.

By pooling three T2DM studies we obtained a pooled safety database of 1026 patients, which allowed a better characterisation of the safety profile of canakinumab than was possible in each individual trial [24-27]. Nevertheless, this sample size was still too small to conclusively evaluate all potential observed safety issues, which may have included spurious chance findings. It also does not allow a proper evaluation of extremely rare events (such as malignancies, neutropenia or renal function). Another key limitation of this pooled safety analysis is that the duration of treatment and follow-up was on average 6 months and only exceeded 1 year in a small subset of patients. This precludes any definite conclusions regarding the long-term safety of canakinumab in patients with T2DM or IGT. However, CANTOS, an ongoing large outcomes study, is evaluating three doses of canakinumab (50, 150 and 300 mg versus placebo) given subcutaneously on a quarterly basis for up to 5 years in order to assess the effects on cardiovascular risk reduction in post-MI patients and will provide long term safety data on canakinumab [20].

In conclusion, this pooled safety analysis over a treatment period of 1.4 years indicates that canakinumab across the evaluated doses in T2DM or IGT patients is safe and well tolerated, adding important information to the overall safety profile of the drug.

## Competing interests

All authors are employees of Novartis and own shares. This work was funded by Novartis.

## Authors’ contributions

AN and CH were primarily responsible for development and review of the clinical study reports for all three studies individually. All authors reviewed the meta-analysis data and provided interpretation of the data. All authors were involved in the development of the manuscript, reviewed and approved the final draft for publication.

## Supplementary Material

Additional file 1: Table S1 Definitions of adverse events of special interest including class effect (IL-1β inhibition) and disease effect (T2DM). Click here for file

Additional file 2: Table S2 Patient demographics and baseline characteristics, by study. Click here for file

Additional file 3: Table S3 Incidence of any adverse events and specified abnormalities in haematology and biochemistry during any time of the post-treatment follow-up period by age subgroup and gender. Click here for file
