# Supplementary material for: Safety and tolerability of canakinumab, an IL-1β inhibitor, in type 2 diabetes mellitus patients: a pooled analysis of three randomised double-blind studies
Source: Cardiovasc Diabetol. 2014 May 17;13:94. doi: 10.1186/1475-2840-13-94 (PMC4033489; doi:10.1186/1475-2840-13-94)
Supplement: Additional file 1: Table S1 — Definitions of adverse events of special interest including class effect (IL-1β inhibition) and disease effect (T2DM). [file 1475-2840-13-94-S1.pdf]

Table A1. Definitions of adverse events of special interest including class effect (IL-1β inhibition) and disease effect (T2DM)

**Infections**		
Primary or secondary SOC 'Infections and infestations'		
**Hypersensitivity/allergy (NMQ)**		
Preferred term 'Acute generalised exanthematous pustulosis'	Preferred term 'Administration site rash'	Preferred term 'Allergic bronchitis'
Preferred term 'Allergic colitis'	Preferred term 'Allergic cough'	Preferred term 'Allergic cystitis'
Preferred term 'Allergic granulomatous angiitis'	Preferred term 'Allergic hepatitis'	Preferred term 'Allergic keratitis'
Preferred term 'Allergic myocarditis'	Preferred term 'Allergic oedema'	Preferred term 'Allergic otitis media'
Preferred term 'Allergic pharyngitis'	Preferred term 'Allergic respiratory disease'	Preferred term 'Allergic respiratory symptom'
Preferred term 'Allergic sinusitis'	Preferred term 'Allergic transfusion reaction'	Preferred term 'Allergy test positive'
Preferred term 'Allergy to vaccine'	Preferred term 'Alveolitis allergic'	Preferred term 'Analgesic asthma syndrome'
Preferred term 'Anaphylactic reaction'	Preferred term 'Anaphylactic shock'	Preferred term 'Anaphylactic transfusion reaction'
Preferred term 'Anaphylactoid reaction'	Preferred term 'Anaphylactoid shock'	Preferred term 'Anaphylaxis treatment'
Preferred term 'Angioedema'	Preferred term 'Antiallergic therapy'	Preferred term 'Antiendomysial antibody positive'
Preferred term 'Anti-neutrophil cytoplasmic antibody positive vasculitis'	Preferred term 'Application site dermatitis'	Preferred term 'Application site hypersensitivity'
Preferred term 'Application site rash'	Preferred term 'Application site urticaria'	Preferred term 'Arthritis allergic'
Preferred term 'Atopy'	Preferred term 'Blepharitis allergic'	Preferred term 'Blood immunoglobulin E abnormal'
Preferred term 'Blood immunoglobulin E increased'	Preferred term 'Bromoderma'	Preferred term 'Bronchospasm'
Preferred term 'Catheter site rash'	Preferred term 'Catheter site urticaria'	Preferred term 'Circumoral oedema'
Preferred term 'Conjunctival oedema'	Preferred term 'Conjunctivitis allergic'	Preferred term 'Contact stomatitis'
Preferred term 'Contrast media allergy'	Preferred term 'Contrast media reaction'	Preferred term 'Corneal oedema'
Preferred term 'Cutaneous vasculitis'	Preferred term 'Dapsone syndrome'	Preferred term 'Dennie-Morgan fold'
Preferred term 'Dermatitis'	Preferred term 'Dermatitis acneiform'	Preferred term 'Dermatitis allergic'
Preferred term 'Dermatitis atopic'	Preferred term 'Dermatitis bullous'	Preferred term 'Dermatitis contact'
Preferred term 'Dermatitis exfoliative'	Preferred term 'Dermatitis exfoliative generalised'	Preferred term 'Dermatitis herpetiformis'
Preferred term 'Dermatitis infected'	Preferred term 'Dermatitis psoriasiform'	Preferred term 'Documented hypersensitivity to administered drug'
Preferred term 'Drug eruption'	Preferred term 'Drug hypersensitivity'	Preferred term 'Drug rash with eosinophilia and systemic symptoms'
Preferred term 'Eczema'	Preferred term 'Eczema infantile'	Preferred term 'Eczema nummular'
Preferred term 'Eczema vaccinatum'	Preferred term 'Eczema vesicular'	Preferred term 'Eczema weeping'
Preferred term 'Encephalitis allergic'	Preferred term 'Encephalopathy allergic'	Preferred term 'Epidermal necrosis'
Preferred term 'Epidermolysis'	Preferred term 'Epidermolysis bullosa'	Preferred term 'Epiglottic oedema'
Preferred term 'Erythema multiforme'	Preferred term 'Erythema nodosum'	Preferred term 'Exfoliative rash'
Preferred term 'Eye allergy'	Preferred term 'Eye oedema'	Preferred term 'Eyelid oedema'
Preferred term 'Face oedema'	Preferred term 'First use syndrome'	Preferred term 'Fixed eruption'
Preferred term 'Giant papillary conjunctivitis'	Preferred term 'Haemorrhagic urticaria'	Preferred term 'Henoch-Schonlein purpura'
Preferred term 'Henoch-Schonlein purpura nephritis'	Preferred term 'Heparin-induced thrombocytopenia'	Preferred term 'Hypersensitivity'
Preferred term 'Immediate post-injection reaction'	Preferred term 'Implant site dermatitis'	Preferred term 'Implant site hypersensitivity'
Preferred term 'Implant site rash'	Preferred term 'Implant site urticaria'	Preferred term 'Infusion site dermatitis'
Preferred term 'Infusion site hypersensitivity'	Preferred term 'Infusion site rash'	Preferred term 'Infusion site urticaria'
Preferred term 'Injection site dermatitis'	Preferred term 'Injection site hypersensitivity'	Preferred term 'Injection site rash'
Preferred term 'Injection site urticaria'	Preferred term 'Injection site vasculitis'	Preferred term 'Interstitial granulomatous dermatitis'
Preferred term 'Iodine allergy'	Preferred term 'Kaposi's varicelliform eruption'	Preferred term 'Kounis syndrome'
Preferred term 'Laryngeal oedema'	'Laryngitis allergic'	Preferred term 'Laryngospasm'
Preferred term 'Laryngotracheal oedema'	Preferred term 'Leukocytoclastic vasculitis'	Preferred term 'Limbal swelling'
Preferred term 'Lip oedema'	Preferred term 'Lip swelling'	Preferred term 'Mucocutaneous rash'
Preferred term 'Multiple allergies'	Preferred term 'Nephritis allergic'	Preferred term 'Nikolsky's sign'
Preferred term 'Oculomucocutaneous syndrome'	Preferred term 'Oculorespiratory syndrome'	Preferred term 'Oedema mouth'
Preferred term 'Oral allergy syndrome'	Preferred term 'Oropharyngeal blistering'	Preferred term 'Oropharyngeal spasm'
Preferred term 'Oropharyngeal swelling'	Preferred term 'Palatal oedema'	Preferred term 'Palpable purpura'
Preferred term 'Periorbital oedema'	Preferred term 'Pharyngeal oedema'	Preferred term 'Photosensitivity allergic reaction'
Preferred term 'Pruritus allergic'	Preferred term 'Radioallergosorbent test positive'	Preferred term 'Rash'
Preferred term 'Rash erythematous'	Preferred term 'Rash follicular'	Preferred term 'Rash generalised'

Preferred term ‘Rash macular’	Preferred term ‘Rash maculo-papular’	Preferred term ‘Rash maculovesicular’
Preferred term ‘Rash morbilliform’	Preferred term ‘Rash neonatal’	Preferred term ‘Rash papulosquamous’
Preferred term ‘Rash pruritic’	Preferred term ‘Rash pustular’	Preferred term ‘Rash rubelliform’
Preferred term ‘Rash scarlatiniform’	Preferred term ‘Rash vesicular’	Preferred term ‘Reaction to azo-dyes’
Preferred term ‘Reaction to colouring’	Preferred term ‘Reaction to drug excipients’	Preferred term ‘Reaction to preservatives’
Preferred term ‘Red man syndrome’	Preferred term ‘Rhinitis allergic’	Preferred term ‘Scleral oedema’
Preferred term ‘Scleritis allergic’	Preferred term ‘Scrotal oedema’	Preferred term ‘Serum sickness’
Preferred term ‘Serum sickness-like reaction’	Preferred term ‘Skin necrosis’	Preferred term ‘Skin reaction’
Preferred term ‘Skin test positive’	Preferred term ‘Small bowel angioedema’	Preferred term ‘Solar urticaria’
Preferred term ‘Solvent sensitivity’	Preferred term ‘Stevens-Johnson syndrome’	Preferred term ‘Swelling face’
Preferred term ‘Swollen tongue’	Preferred term ‘Tongue oedema’	Preferred term ‘Toxic epidermal necrolysis’
Preferred term ‘Toxic skin eruption’	Preferred term ‘Tracheal oedema’	Preferred term ‘Type I hypersensitivity’
Preferred term ‘Type II hypersensitivity’	Preferred term ‘Type III immune complex mediated reaction’	Preferred term ‘Type IV hypersensitivity reaction’
Preferred term ‘Urticaria’	Preferred term ‘Urticaria cholinergic’	Preferred term ‘Urticaria chronic’
Preferred term ‘Urticaria contact’	Preferred term ‘Urticaria papular’	Preferred term ‘Urticaria physical’
Preferred term ‘Urticaria pigmentosa’	Preferred term ‘Urticaria vesiculosa’	Preferred term ‘Vaccination site dermatitis’
Preferred term ‘Vaccination site exfoliation’	Preferred term ‘Vaccination site hypersensitivity’	Preferred term ‘Vaccination site rash’
Preferred term ‘Vaccination site urticaria’	Preferred term ‘Vaccination site vesicles’	Preferred term ‘Vaginal exfoliation’
Preferred term ‘Vaginal ulceration’	Preferred term ‘Vasculitic rash’	Preferred term ‘Vulval ulceration’
Preferred term ‘Vulvovaginal ulceration’	Preferred term ‘Acute haemolytic transfusion reaction’	Preferred term ‘Acute respiratory failure’
Preferred term ‘Allergy to chemicals’	Preferred term ‘Allergy to fermented products’	Preferred term ‘Alpha tumour necrosis factor increased’
Preferred term ‘Alveolitis’	Preferred term ‘Antibody test abnormal’	Preferred term ‘Antibody test positive’
Preferred term ‘Anti-insulin antibody increased’	Preferred term ‘Anti-insulin antibody positive’	Preferred term ‘Anti-insulin receptor antibody increased’
Preferred term ‘Anti-insulin receptor antibody positive’	Preferred term ‘Asthma’	Preferred term ‘Asthma late onset’
Preferred term ‘Asthmatic crisis’	Preferred term ‘Auricular swelling’	Preferred term ‘Blister’
Preferred term ‘Blood immunoglobulin A abnormal’	Preferred term ‘Blood immunoglobulin A increased’	Preferred term ‘Blood immunoglobulin D increased’
Preferred term ‘Blood immunoglobulin G abnormal’	Preferred term ‘Blood immunoglobulin G increased’	Preferred term ‘Blood immunoglobulin M abnormal’
Preferred term ‘Blood immunoglobulin M increased’	Preferred term ‘Bronchial hyperreactivity’	Preferred term ‘Bronchial oedema’
Preferred term ‘Bullous impetigo’	Preferred term ‘Capillaritis’	Preferred term ‘Charcot-Leyden crystals’
Preferred term ‘Choking’	Preferred term ‘Choking sensation’	Preferred term ‘Chronic eosinophilic rhinosinusitis’
Preferred term ‘Chronic hyperplastic eosinophilic sinusitis’	Preferred term ‘Circulatory collapse’	Preferred term ‘Complement factor C1 decreased’
Preferred term ‘Complement factor C1 increased’	Preferred term ‘Complement factor C2 decreased’	Preferred term ‘Complement factor C2 increased’
Preferred term ‘Complement factor C3 decreased Complement factor C4 increased’	Preferred term ‘Complement factor C3 increased Complement factor decreased’	Preferred term ‘Complement factor C4 decreased Complement factor increased’
Preferred term ‘Complement fixation abnormal’	Preferred term ‘Complement fixation test positive’	Preferred term ‘Conjunctivitis’
Preferred term ‘Corneal exfoliation’	Preferred term ‘Delayed haemolytic transfusion reaction’	Preferred term ‘Delayed serologic transfusion reaction’
Preferred term ‘Drug-induced liver injury’	Preferred term ‘Eosinophil count abnormal’	Preferred term ‘Eosinophil count increased’
Preferred term ‘Eosinophil percentage abnormal’	Preferred term ‘Eosinophil percentage increased’	Preferred term ‘Eosinophilia’
Preferred term ‘Eosinophilia myalgia syndrome’	Preferred term ‘Eosinophilic bronchitis’	Preferred term ‘Eosinophilic oesophagitis’
Preferred term ‘Eosinophilic pneumonia’	Preferred term ‘Eosinophilic pneumonia acute’	Preferred term ‘Eosinophilic pneumonia chronic’
Preferred term ‘Erythema’	Preferred term ‘Eye swelling’	Preferred term ‘Flushing’
Preferred term ‘Gastrointestinal oedema’	Preferred term ‘Generalised erythema’	Preferred term ‘Generalised oedema’
Preferred term ‘Genital rash’	Preferred term ‘Genital swelling’	Preferred term ‘Genital ulceration’
Preferred term ‘Gingival oedema’	Preferred term ‘Gingival swelling’	Preferred term ‘Gleich’s syndrome’
Preferred term ‘Haemolytic transfusion reaction’	Preferred term ‘Hereditary angioedema’	Preferred term ‘HLA marker study positive’
Preferred term ‘Hypotensive transfusion reaction idiopathic urticaria’	Preferred term ‘Immune complex level increased’	Preferred term ‘Immunoglobulins abnormal’
Preferred term ‘Immunoglobulins increased’	Preferred term ‘Immunology test abnormal’	Preferred term ‘Infantile asthma’
Preferred term ‘Interstitial lung disease’	Preferred term ‘Laryngeal dyspnoea’	Preferred term ‘Laryngeal obstruction’
Preferred term ‘Leukotriene increased’	Preferred term ‘Lip exfoliation’	Preferred term ‘Localised oedema’
Preferred term ‘Mechanical urticaria’	Preferred term ‘Mesenteric panniculitis’	Preferred term ‘Mouth ulceration’
Preferred term ‘Mucocutaneous ulceration’	Preferred term ‘Mucosa vesicle’	Preferred term ‘Mucosal erosion’
Preferred term ‘Mucosal exfoliation’	Preferred term ‘Mucosal necrosis’	Preferred term ‘Mucosal ulceration’
Preferred term ‘Necrotising panniculitis’	Preferred term ‘Neurodermatitis’	Preferred term ‘Neutralising antibodies positive’
Preferred term ‘Non-neutralising antibodies positive’	Preferred term ‘Occupational asthma’	Preferred term ‘Oedema mucosal’
Preferred term ‘Oral mucosal exfoliation’	Preferred term ‘Orbital oedema’	Preferred term ‘Panniculitis’

Preferred term ‘Penile exfoliation’	Preferred term ‘Penile oedema’	Preferred term ‘Penile swelling’
Preferred term ‘Perivascular dermatitis’	Preferred term ‘Pneumonitis’	Preferred term ‘Post transfusion purpura’
Preferred term ‘Prurigo’	Preferred term ‘Pruritus’	Preferred term ‘Pruritus generalised’
Preferred term ‘Pulmonary eosinophilia’	Preferred term ‘Reactive airways dysfunction syndrome’	Preferred term ‘Respiratory arrest’
Preferred term ‘Respiratory distress’	Preferred term ‘Respiratory failure’	Preferred term ‘Respiratory tract oedema’
Preferred term ‘Reversible airways obstruction’	Preferred term ‘Rhinitis perennial’	Preferred term ‘Rhinitis seasonal’
Preferred term ‘Scrotal swelling’	Preferred term ‘Seasonal allergy’	Preferred term ‘Septal panniculitis’
Preferred term ‘Shock’	Preferred term ‘Skin erosion’	Preferred term ‘Skin exfoliation’
Preferred term ‘Skin oedema’	Preferred term ‘Skin swelling’	Preferred term ‘Sneezing’
Preferred term ‘Status asthmaticus’	Preferred term ‘Stomatitis’	Preferred term ‘Streptokinase antibody increased’
Preferred term ‘Stridor’	Preferred term ‘Suffocation feeling’	Preferred term ‘Throat tightness’
Preferred term ‘Tongue exfoliation’	Preferred term ‘Tracheal obstruction’	Preferred term ‘Tracheostomy’
Preferred term ‘Upper airway obstruction’	Preferred term ‘Vaginal oedema’	Preferred term ‘Visceral oedema’
Preferred term ‘Vulval oedema’	Preferred term ‘Vulvovaginal rash’	Preferred term ‘Vulvovaginal swelling’
Preferred term ‘Wheezing’		
**Thrombocytopenia**		
Narrow ‘Haemorrhages SMQ’	Narrow ‘Haematopoietic thrombocytopenia SMQ’	
**Hypertension**		
Preferred term ‘Accelerated hypertension’	Preferred term ‘Blood pressure ambulatory increased’	Preferred term ‘Blood pressure diastolic increased’
Preferred term ‘Blood pressure inadequately controlled’	Preferred term ‘Blood pressure increased’	Preferred term ‘Blood pressure systolic increased’
Preferred term ‘Diastolic hypertension’	Preferred term ‘Eclampsia’	Preferred term ‘Endocrine hypertension’
Preferred term ‘Essential hypertension’	Preferred term ‘Gestational hypertension’	Preferred term ‘Hyperaldosteronism’
Preferred term ‘Hypertension’	Preferred term ‘Hypertensive crisis’	Preferred term ‘Hypertensive emergency’
Preferred term ‘Labile hypertension’	Preferred term ‘Malignant hypertension’	Preferred term ‘Malignant hypertensive heart disease’
Preferred term ‘Malignant renal hypertension’	Preferred term ‘Maternal hypertension affecting foetus’	Preferred term ‘Mean arterial pressure increased’
Preferred term ‘Neurogenic hypertension’	Preferred term ‘Orthostatic hypertension’	Preferred term ‘Pre-eclampsia’
Preferred term ‘Renal hypertension’	Preferred term ‘Renovascular hypertension’	Preferred term ‘Retinopathy hypertensive’
Preferred term ‘Secondary hypertension’	Preferred term ‘Systolic hypertension’	
**Major adverse cardiovascular events (MACE)**		
Broad ‘Cardiac failure (SMQ)’	Broad ‘Ischaemic heart disease (SMQ)’ without preferred term ‘Angina pectoris’	Broad ‘Cerebrovascular disorders (SMQ)’ without preferred term ‘Carotid arteriosclerosis’
Broad ‘Embolic and thrombotic events (SMQ)’ but only if the preferred term is ‘Ischaemic cerebral infarction’ or ‘Myocardial infarction’		
**Hepatic disorders**		
Narrow ‘Hepatic disorders (SMQ)’		
**Vertigo**		
Primary or secondary HLT ‘Vertigos NEC’	Preferred term ‘Dizziness’	Preferred term ‘Dizziness postural’
Preferred term ‘Balance disorder’	Preferred term ‘Dizziness exertional’	
**Injection site reactions**		
Primary or secondary HLGT ‘Administration site reactions’	Preferred term ‘Immediate post-injection reaction’	
Hypoglycaemia - The ‘Hypoglycaemia’ Novartis MedDRA query definition includes only the narrow terms below and does not include symptoms of hypoglycaemia		
Preferred term ‘Blood glucose decreased’	Preferred term ‘Hypoglycaemia’	Preferred term ‘Hypoglycaemia neonatal’
Preferred term ‘Hypoglycaemia unawareness’	Preferred term ‘Hypoglycaemic coma’	Preferred term ‘Hypoglycaemic encephalopathy’
Preferred term ‘Hypoglycaemic seizure’	Preferred term ‘Hypoglycaemic unconsciousness’	Preferred term ‘Neuroglycopenia’
Preferred term ‘Shock hypoglycaemic’		
**Changes in renal function**		
Primary or secondary HLT ‘Renal failure and impairment’	Primary or secondary HLT ‘Nephropathies and tubular disorders NEC’	Broad ‘Acute renal failure (SMQ)’
**Lymphoid organ toxicity**		
Primary or secondary HLGT ‘Spleen, lymphatic and reticuloendothelial system disorders’	Preferred term ‘Lymphoid hyperplasia of appendix’	Preferred term ‘Tonsillar hypertrophy’
**Malignancies**		
Narrow ‘Malignancies (SMQ)’		
**Neutropenia**		
Broad ‘Neutropenia (SMQ)’	Primary or secondary HLT ‘Neutropenias’	

All coding was based on Medical Dictionary for Drug Regulatory Activities (MedDRA) version 15.0.
NEC, Not elsewhere classified; HLT, high level term; HLGT, high level group term; SOC; system organ class; SMQ, Standardized MedDRA Query. The narrow version of an SMQ contains only those terms that are highly likely to represent the condition of interest, while the broad version also includes terms that are not in all instances indicative of the condition of interest.
