# Supplementary material for: Safety and tolerability of canakinumab, an IL-1β inhibitor, in type 2 diabetes mellitus patients: a pooled analysis of three randomised double-blind studies
Source: Cardiovasc Diabetol. 2014 May 17;13:94. doi: 10.1186/1475-2840-13-94 (PMC4033489; doi:10.1186/1475-2840-13-94)
Supplement: Additional file 2: Table S2 — Patient demographics and baseline characteristics, by study. [file 1475-2840-13-94-S2.pdf]

Table A2. Patient demographics and baseline characteristics, by study

	Study 1 (CACZ885A2213)[27]										Study 2 (CACZ885I2202)[24, 25]					Study 3 (CACZ885I2207)[26]				
	Cohort 1		Cohort 2		Cohort 3			Cohort 4								T2DM		IGT		
	Placebo (N=5)	Intermediate dose (N=10)	Placebo (N=45)	High dose (N=45)	Placebo (N=24)	Intermediate dose (N=49)	Medium dose (N=23)	Placebo (N=10)	Low dose (N=20)	Total (N=231)	Placebo (N=179)	Intermediate dose (N=188)	Medium dose (N=92)	High dose (N=92)	Total (N=551)	Placebo (N=65)	Medium dose (N=125)	Placebo (N=26)	Medium dose (N=28)	Total (N=244)
Age (years)	52±6.96	53±7.6	57.5±7.95	57.1±9.38	54.7±6.41	54.4±7.95	55.1±9.28	56±8.67	53.9±9.07	55.6±8.35	54.3±10.15	54.5±10.01	53±9.29	53.7±10.36	54.1±9.99	57.3±10.08	58.5±9.89	57.3±10.15	52.8±10.9	57.4±10.18
Age group (years), n (%)																				
<65	5 (100)	9 (90.0)	35 (77.8)	36 (80.0)	23 (95.8)	42 (85.7)	20 (87.0)	9 (90.0)	17 (85.0)	196 (84.8)	148 (82.7)	155 (82.4)	79 (85.9)	73 (79.3)	455 (82.6)	46 (70.8)	86 (68.8)	19 (73.1)	25 (89.3)	176 (72.1)
≥65	0	1 (10.0)	10 (22.2)	9 (20.0)	1 (4.2)	7 (14.3)	3 (13.0)	1 (10.0)	3 (15.0)	35 (15.2)	31 (17.3)	33 (17.6)	13 (14.1)	19 (20.7)	96 (17.4)	19 (29.2)	39 (31.2)	7 (26.9)	3 (10.7)	68 (27.9)
Sex, n (%)																				
Male	2 (40.0)	4 (40.0)	31 (68.9)	24 (53.3)	8 (33.3)	19 (38.8)	9 (39.1)	8 (80.0)	10 (50.0)	115 (49.8)	105 (58.7)	104 (55.3)	45 (48.9)	57 (62.0)	311 (56.4)	38 (58.5)	68 (54.4)	11 (42.3)	16 (57.1)	133 (54.5)
Female	3 (60.0)	6 (60.0)	14 (31.1)	21 (46.7)	16 (66.7)	30 (61.2)	14 (60.9)	2 (20.0)	10 (50.0)	116 (50.2)	74 (41.3)	84 (44.7)	47 (51.1)	35 (38.0)	240 (43.6)	27 (41.5)	57 (45.6)	15 (57.7)	12 (42.9)	111 (45.5)
Race, n (%)																				
Caucasian	4 (80.0)	9 (90.0)	43 (95.6)	42 (93.3)	21 (87.5)	46 (93.9)	22 (95.7)	10 (100)	19 (95.0)	216 (93.5)	73 (40.8)	79 (42.0)	39 (42.4)	39 (42.4)	230 (41.7)	50 (76.9)	89 (71.2)	17 (65.4)	18 (64.3)	174 (71.3)
Black	0	1 (10.0)	1 (2.2)	2 (4.4)	3 (12.5)	3 (6.1)	1 (4.3)	0	1 (5.0)	12 (5.2)	9 (5.0)	8 (4.3)	5 (5.4)	3 (3.3)	25 (4.5)	1 (1.5)	1 (0.8)	0	1 (3.6)	3 (1.2)
Asian	0	0	0	0	0	0	0	0	0	0	75 (41.9)	81 (43.1)	39 (42.4)	41 (44.6)	236 (42.8)	14 (21.5)	29 (23.2)	8 (30.8)	9 (32.1)	60 (24.6)
Others	1 (20.0)	0	1 (2.2)	1 (2.2)	0	0	0	0	0	3 (1.3)	22 (12.3)	20 (10.6)	9 (9.8)	9 (9.8)	60 (10.9)	0	**6 (4.8)**	1 (3.8)	0	7 (2.9)
BMI (kg/m²), n (%)																				
<30	2 (40.0)	5 (50.0)	17 (37.8)	21 (46.7)	9 (37.5)	16 (32.7)	7 (30.4)	7 (70.0)	9 (45.0)	93 (40.3)	104 (58.1)	110 (58.5)	44 (47.8)	51 (55.4)	309 (56.1)	29 (44.6)	52 (41.6)	15 (57.7)	12 (42.9)	108 (44.3)
≥30	3 (60.0)	5 (50.0)	28 (62.2)	24 (53.3)	15 (62.5)	33 (67.3)	16 (69.6)	3 (30.0)	11 (55.0)	138 (59.7)	75 (41.9)	78 (41.5)	48 (52.2)	41 (44.6)	242 (43.9)	36 (55.4)	73 (58.4)	11 (42.3)	16 (57.1)	136 (55.7)
HbA1c (%)	7.1±0.66	8.0±0.79	7.8±0.82	7.9±0.68	7.6±0.89	7.5±0.7	7.7±0.75	8±1.08	7.6±0.79	7.7±0.78	7.5±0.8	7.4±0.81	7.4±0.78	7.5± 0.76	7.4±0.79	7.2±0.46	7.1±0.44	6.1±0.41	6.1±0.65	6.9±0.64
FPG (mmol/L)	8.9±2.6	9.7±1.58	8.9±1.68	9.0±1.62	8.8±1.5	9.1±2.15	8.9±1.86	9.2±2.22	8.8±1.84	9.0±1.81	7.8±1.66	7.6±1.62	7.8±1.79	7.7±1.34	7.7±1.62	8.2±2.12	7.8±1.85	5.8±0.65	5.9±0.6	7.5±1.94
Duration of type 2 diabetes (years)	8.6±3.4	6.8±7.27	6.2±5.14	7.9±5.29	5.9±4.93	7.3±4.76	5.3±4.59	10.6±5.53	5.0±3.62	6.8±5.08	4.0±4.46	3.8±4.15	3.4±3.91	3.2±3.51	3.7±4.11	8.5±6.6	9.9±7.59	NA	NA	9.4±7.28
Subjects with impaired glucose tolerance[1]	0	0	0	0	0	0	0	0	0	0	0	0	0	0	0	0	0	26	28	54
Diabetes complications, n (%)[2]	0	0	0	0	0	0	0	0	0	0	18 (10.1)	28 (14.9)	8 (8.7)	16 (17.4)	70 (12.7)	12 (18.5)	28 (22.4)	0	0	40 (21.1)
Retinopathy											6 (3.4)	3 (1.6)	3 (3.3)	1 (1.1)	13 (2.4)	2 (3.1)	7 (5.6)	0	0	9 (4.7)
Neuropathy											10 (5.6)	25 (13.3)	4 (4.3)	11 (12.0)	50 (9.1)	9 (13.8)	23 (18.4)	0	0	32 (16.8)
Nephropathy											4 (2.2)	2 (1.1)	0	4 (4.3)	10 (1.8)	3 (4.6)	8 (6.4)	0	0	11 (5.8)
Use of statins, n (%)[2]																				
Yes	0	0	4 (8.9)	6 (13.3)	4 (16.7)	2 (4.1)	2 (8.7)	1 (10.0)	2 (10.0)	21 (9.1)	43 (24.0)	62 (33.0)	25 (27.2)	26 (28.3)	156 (28.3)	28 (43.1)	69 (55.2)	7 (26.9)	8 (28.6)	112 (45.9)
No	5 (100)	10 (100)	41 (91.1)	39 (86.7)	20 (83.3)	47 (95.9)	21 (91.3)	9 (90.0)	18 (90.0)	210 (90.9)	136 (76.0)	126 (67.0)	67 (72.8)	66 (71.7)	395 (71.7)	37 (56.9)	56 (44.8)	19 (73.1)	20 (71.4)	132 (54.1)

Data are expressed as mean±standard deviation (SD), unless otherwise stated.
BMI, body mass index; FPG, fasting plasma glucose; HbA1c, glycosylated haemoglobin A1c
Low dose: 0.03 mg/kg i.v. once; intermediate dose: 0.1 and 0.3 mg/kg i.v. once, 5 and 15 mg s.c. monthly; medium dose: 1.5 mg/kg i.v. once, 50 mg s.c. monthly and 150 mg s.c. once; high dose: 10 mg/kg i.v. once and 150 mg s.c. monthly.
[1]Pre-diabetic subjects with impaired glucose tolerance were enrolled only in study CACZ885I2207.
[2]Information on cardiovascular risk/ disease factors and diabetes complications was not collected in study CACZ885A2213.
