# Supplementary material for: Safety and tolerability of canakinumab, an IL-1β inhibitor, in type 2 diabetes mellitus patients: a pooled analysis of three randomised double-blind studies
Source: Cardiovasc Diabetol. 2014 May 17;13:94. doi: 10.1186/1475-2840-13-94 (PMC4033489; doi:10.1186/1475-2840-13-94)
Supplement: Additional file 3: Table S3 — Incidence of any adverse events and specified abnormalities in haematology and biochemistry during any time of the post-treatment follow-up period by age subgroup and gender. [file 1475-2840-13-94-S3.pdf]

Table A3. Incidence of any adverse events and specified abnormalities in haematology and biochemistry during any time of the post-treatment follow-up period by age subgroup and gender

Parameters	Canakinumab pooled doses																Placebo			
	Low dose (mg/kg)				Intermediate dose (mg/kg)				Medium dose (mg/kg)				High dose (mg/kg)							
	By age (years)		By gender		By age (years)		By gender		By age (years)		By gender		By age (years)		By gender		By age (years)		By gender	
	<65	≥65	Male	Female	<65	≥65	Male	Female	<65	≥65	Male	Female	<65	≥65	Male	Female	<65	≥65	Male	Female
Total AEs n (%)	8 (47.1)	2 (66.7)	3 (30.0)	7 (70.0)	86 (41.7)	21 (51.2)	58 (45.7)	49 (40.8)	81 (38.6)	22 (37.9)	49 (35.5)	54 (41.5)	54 (49.5)	13 (46.4)	32 (39.5)	35 (62.5)	106 (37.2)	28 (40.6)	75 (36.9)	59 (39.1)
IR (%)	14.48	33.04	8.39	27.39	9.8	12.88	10.41	10.13	15.53	21.1	15.46	17.48	12.89	9.75	8.71	18.96	10.76	15.12	10.47	12.98
Total SAEs n (%)	0	0	0	0	3 (1.5)	1 (2.4)	4 (3.1)	0	5 (2.4)	0	3 (2.2)	2 (1.5)	6 (5.5)	2 (7.1)	6 (7.4)	2 (3.6)	10 (3.5)	0	7 (3.4)	3 (2.0)
IR (%)					0.24	0.34	0.5		0.66		0.68	0.43	0.9	1.09	1.21	0.56	0.71		0.71	0.42
Discontinuations due to AEs n (%)	0.0000	1 (33.3)	1 (10.0)	0.0000	1 (0.5)	0.0000	0.0000	1 (0.8)	0.0000	0.0000	0.0000	0.0000	1 (0.9)	1 (3.6)	0.0000	2 (3.6)	1 (0.4)	0.0000	0.0000	1 (0.7)
IR (%)		8.6900	2.2000		0.0800			0.1300					0.1400	0.5400		0.5500	0.0700			0.1400
**Haematology**																				
WBC (total) any grade 1–4, n	0.0000	0.0000	0.0000	0.0000	18.0000	1.0000	11.0000	8.0000	16.0000	2.0000	5.0000	13.0000	8.0000	2.0000	7.0000	3.0000	11.0000	3.0000	8.0000	6.0000
IR (%)	0.0000	0.0000	0.0000	0.0000	2.4900	0.6100	2.5500	1.7600	3.5100	1.9400	1.8000	4.6300	1.8500	1.8700	2.2700	1.3000	1.1900	1.5300	1.2100	1.3000
Dose trend test, P value									0.0796	0.7443	0.3124	0.1503								
Absolute neutrophils, any grade 1–4, n	1.0000	0.0000	0.0000	1.0000	17.0000	4.0000	10.0000	11.0000	25.0000	2.0000	13.0000	14.0000	9.0000	4.0000	7.0000	6.0000	9.0000	1.0000	5.0000	5.0000
IR (%)	1.0300	0.0000	0.0000	1.7900	2.6200	2.9400	2.6300	2.7200	5.6300	1.9600	5.0600	4.8400	3.9000	7.8400	3.9800	5.6600	1.3300	0.7100	1.0600	1.4400
Dose trend test, P value									0.0006	0.0591	0.0053	0.0088								
Platelet count (direct), any grade 1–4, n	1.0000	0.0000	1.0000	0.0000	10.0000	1.0000	7.0000	4.0000	8.0000	1.0000	5.0000	4.0000	4.0000	0.0000	2.0000	2.0000	7.0000	4.0000	8.0000	3.0000
IR (%)	1.0600	0.0000	2.0000	0.0000	1.3600	0.6100	1.6100	0.8600	1.7000	0.9900	1.8200	1.3500	0.9200	0.0000	0.6300	0.8600	0.7600	2.1200	1.2500	0.6400
Dose trend test, P value									0.4689	0.0910	0.6030	0.4930								
**Biochemistry**																				
SGOT (AST) (U/L), any grade 1–4, n	0.0000	0.0000	0.0000	0.0000	1.0000	0.0000	0.0000	1.0000	0.0000	0.0000	0.0000	0.0000	2.0000	0.0000	0.0000	2.0000	0.0000	0.0000	0.0000	0.0000
IR (%)	0.0000	0.0000	0.0000	0.0000	0.1300	0.0000	0.0000	0.2100	0.0000	0.0000	0.0000	0.0000	0.4400	0.0000	0.0000	0.8300	0.0000	0.0000	0.0000	0.0000
Dose trend test, P value									0.0758	NA	NA	0.0783								
SGPT (ALT) (U/L), any grade 1–4, n	0.0000	0.0000	0.0000	0.0000	2.0000	0.0000	1.0000	1.0000	4.0000	0.0000	1.0000	3.0000	4.0000	1.0000	1.0000	4.0000	2.0000	0.0000	2.0000	0.0000
IR (%)	0.0000	0.0000	0.0000	0.0000	0.2600	0.0000	0.2200	0.2100	0.8400	0.0000	0.3600	1.0000	0.8800	0.8800	0.3000	1.6500	0.2100	0.0000	0.3000	0.0000
Dose trend test, P value									0.0358	0.1221	0.9835	0.0017								
Bilirubin (total) (umol/L), any grade 1–4, n	0.0000	0.0000	0.0000	0.0000	0.0000	0.0000	0.0000	0.0000	3.0000	0.0000	2.0000	1.0000	3.0000	1.0000	4.0000	0.0000	6.0000	0.0000	5.0000	1.0000
IR (%)	0.0000	0.0000	0.0000	0.0000	0.0000	0.0000	0.0000	0.0000	0.6300	0.0000	0.7100	0.3300	0.6600	0.8500	1.2600	0.0000	0.6300	0.0000	0.7500	0.2100
Dose trend test, P value									0.9610	0.1218	0.5194	0.7900								

Incidence rate (IR) is expressed as % per month for AEs and % per visit for laboratory abnormalities. WBC, white blood cell; SGOT, serum glutamic oxaloacetic transaminase; SGPT, serum glutamic pyruvic transaminase.

Low dose: 0.03 mg/kg i.v. once; intermediate dose: 0.1 and 0.3 mg/kg i.v. once, 5 and 15 mg s.c. monthly; medium dose: 1.5 mg/kg i.v. once, 50 mg s.c. monthly and 150 mg s.c. once; high dose: 10 mg/kg i.v. once and 150 mg s.c. monthly. Total WBC count: Normal range=4.5–11.0´10^9/L; Absolute neutrophil counts: Normal range=1.5–8.0´10^9/L; Haemoglobin: Normal range=13.5–17.5 % (males) and 12.0–16.0 % (females); Platelet count (direct): Normal range=150–450´10^9/L; Absolute lymphocyte count: Normal range=1.0–4.0´10^9/L; SGOT (AST): Normal range=3–44 U/L; SGPT (ALT): Normal range=0–40 U/L; Bilirubin (total): Normal range=5–17.0 µmol/L; Alkaline phosphatase, serum: Normal range=30–125 U/L; Creatinine: Normal range=45–90 µmol/L (females) and 60–110 µmol/L (males).
